# Helminths of veterinary and zoonotic importance in Nigerian ruminants: a 46-year meta-analysis (1970–2016) of their prevalence and distribution

**DOI:** 10.1186/s40249-018-0438-z

**Published:** 2018-05-29

**Authors:** Solomon Ngutor Karshima, Beatty-Viv Maikai, Jacob Kwada Paghi Kwaga

**Affiliations:** 1 Department of Veterinary Public Health and Preventive Medicine, University of Jos, PMB, 2084 Jos, Nigeria; 2 Department of Veterinary Public Health and Preventive Medicine, Ahmadu Bello University, PMB, 1045 Zaria, Nigeria

**Keywords:** Cestodes, Geographical distribution, Helminths, Nematodes, Nigeria, Prevalence, Ruminants, Trematodes

## Abstract

**Background:**

The livestock industry plays a vital role in the economy of Nigeria. It serves as a major source of income and livelihood for majority of Nigerians who are rural settlers and contributes about 5.2% to the National Gross Domestic Product (GDP). Helminths however, cause economic losses due to reductions in milk production, weight gain, fertility and carcass quality. Zoonotic helminths of livestock origin cause health problems in humans.

**Methods:**

Using the Preferred Reporting Items for Systematic Review and Meta-Analysis (PRISMA) guidelines, the prevalence and distribution of helminths of veterinary and zoonotic importance in Nigerian ruminants were determined in a meta-analysis of data published between 1970 and 2016. Data were stratified based on regions, hosts, study periods, sample sizes and study types while helminths were phylogenetically grouped into cestodes, nematodes and trematodes.

**Results:**

Data from 44 studies reported across 19 Nigerian states revealed an overall pooled prevalence estimate (PPE) of 7.48% (95% *CI*: 7.38–7.57) for helminths of veterinary and zoonotic importance from a total of 320 208 ruminants. We observed a significant variation (*P* < 0.001) between the PPEs range of 1.90% (95% *CI*: 1.78–2.02) and 60.98% (95% *CI*: 58.37–63.55) reported across different strata. High heterogeneity (99.78, 95% *CI*: 7.38–7.57) was observed. *Strongyloides papillosus* was the most prevalent (Prev: 32.02%, 95% *CI*: 31.01–33.11), while, *Fasciola gigantica* had the widest geographical distribution.

**Conclusions:**

Helminths of veterinary and zoonotic importance are prevalent in ruminants and well distributed across Nigeria. Our findings show that helminths of ruminants may also be possible causes of morbidity in humans and economic losses in the livestock industry in Nigeria. High heterogeneity was observed within studies and the different strata. Good agricultural practices on farms, standard veterinary meat inspection and adequate hygiene and sanitation in abattoirs, farms and livestock markets need to be implemented in Nigeria in order to reduce the economic, public health and veterinary threats due to these helminths.

**Electronic supplementary material:**

The online version of this article (10.1186/s40249-018-0438-z) contains supplementary material, which is available to authorized users.

## Multilingual abstracts

Please see Additional file 1 for translations of the abstract into the five official working languages of the United Nations.

## Background

Helminths of ruminants refer to a group of complex multicellular eukaryotic parasites which are infective to animals and humans in which case they are called zoonoses [1]. This group of parasites cause serious economic and public health problems in many resource-limited countries across the globe. In Nigeria for instance, these problems are influenced by inadequate veterinary and medical care as well as inadequate policies on disease control among many other factors [2].

Helminth parasites of ruminants are broadly grouped into two phyla, namely nemathelminthes which are nematodes or roundworms such as *Haemonchus*, *Bonostomum*, *Oesophagostomum* and *Chabertia* and platyhelminthes which include cestodes (e.g. *Avitellina*, *Moniezia*, *Stilesia* and *Taenia*) and trematodes such as *Dicrocoelium*, *Eurytrema*, *Fasciola* and *Paramphistomum* [3]. Transmission of these parasites may be through the ingestion of parasitic eggs and infective larvae on contaminated pasture, water, soil, human hands or tissues of infected vertebrate intermediate hosts, skin penetration, transplacental as well as arthropod and gastropod intermediate hosts [4]. Transmission is influenced by factors including poor hygiene and sanitation, indiscriminate and open defecation [5], as well as environmental factors like temperature, humidity, rainfall [6] and soil moisture [7]. Lack of strategic de-worming of livestock [8, 9], poverty and overcrowding [10] are additional factors.

The negative impacts of helminths on livestock productivity still remain a major challenge in the livestock industry globally [11] despite the projected increased dependence on agriculture in the nearest future [12]. These parasites cause serious economic losses in ruminants ranging from growth rate decrease and poor quality of skin and hides to reductions in the production of milk, meat and wool [13]. For instance, evidence revealed that lactating cows may lose 294.8 kg of milk on average per lactation due to helminth parasites [14, 15]. In Nigeria, infection prevalence rates range between 25.6 and 91.4% [16–19]. Economic losses caused by the rejection of editable organs of slaughtered food animals during veterinary meat inspections were also documented [20–22].

From the public health point of view, reports of zoonotic meta-cestodes; *Cysticercus bovis* and hydatid cyst [19, 23, 24], nematode; *Oesophagostomum* [25–27] and trematodes; *Dicrocoelium dendriticum*, *Eurytrema pancreaticum* and *Fasciola gigantica* [22, 28] entering the food chain in Nigeria are of great public health concern. Human infections with these parasites may result in diarrhoea, retarded growth, intellectual and cognitive retardation [29], cystic echinococcosis and cysticercosis [30].

The livestock industry plays a vital role in the economy of Nigeria. It serves as a major source of income and livelihood for majority of Nigerians who are rural settlers and contributes about 5.2% of the National Gross Domestic Product (GDP) [31]. In addition, cattle, sheep and goats contribute over 80% of the total meat produced in Nigeria [25, 32]. Despite these benefits, helminth infections still cause serious economic losses in Nigeria as a result of reductions in milk production, weight gain, fertility and carcass quality. The aim of this study was to provide epidemiological information which will help in instituting sustainable control programmes against these parasites, thus reducing economic losses associated with these helminths and maximising the contribution of the livestock industry to Nigeria’s GDP.

## Methods

### Study areas

**Fig. 1:**
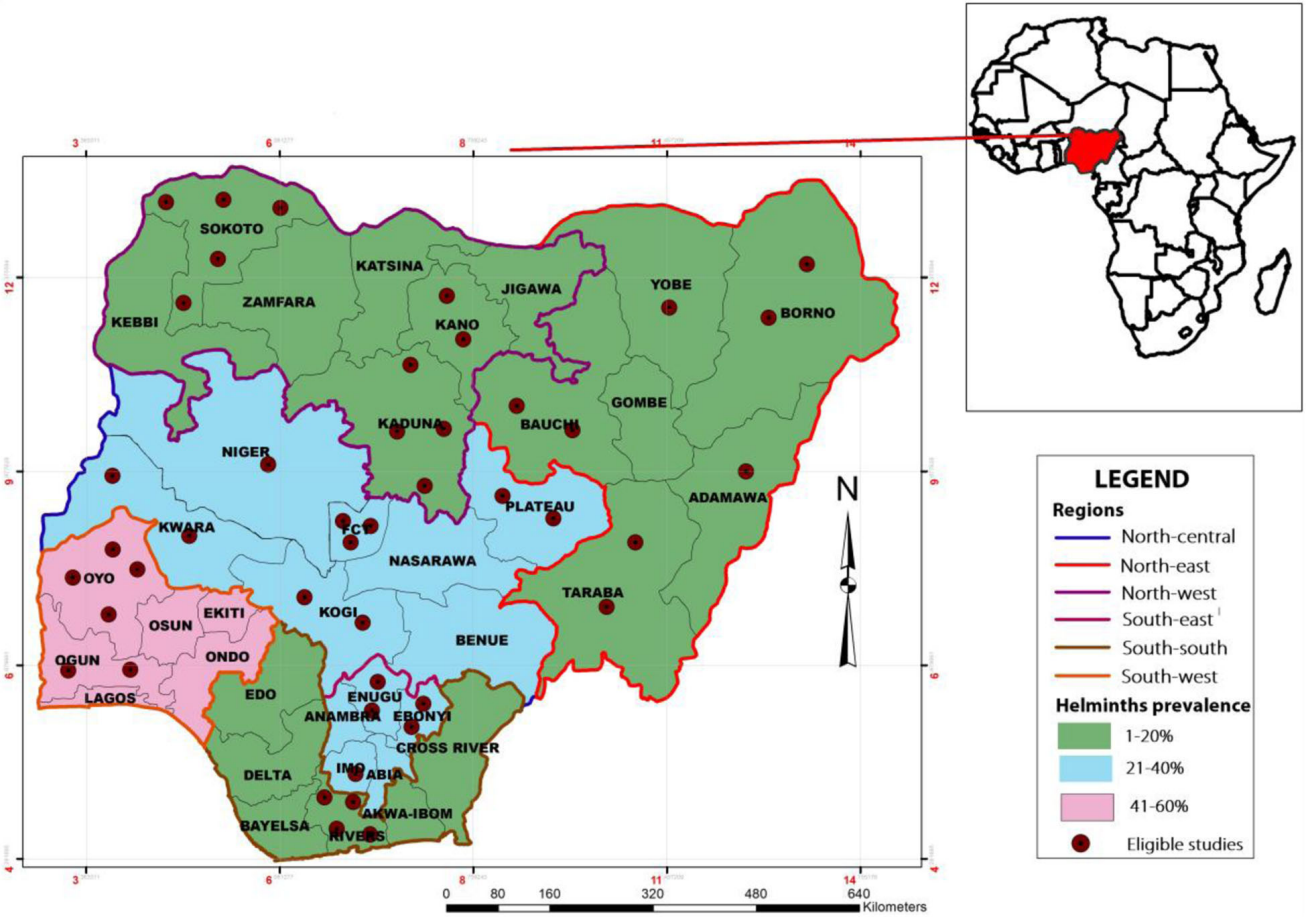
Distribution of eligible studies and regional prevalence of helminths in ruminants in Nigeria

We included in the present review studies published on helminths of veterinary and zoonotic importance in ruminants from Nigeria (West Africa; 4–14 °N; 3–14 °E) which covers a surface area totalling 923 768 km^2^ (Fig. 1). In Nigeria, there are two seasons; the rainy season which runs from March to November in the Southern region and May to October in the Northern region, as well as the dry season which runs from December to February in the South and November to April in the North [33].

### Bibliographic search strategy

The study followed the Preferred Reporting Items for Systematic Review and Meta-Analysis (PRISMA) guidelines published by Moher et al. [34], and the three authors conducted independently the literature search. We used the PRISMA checklist (Additional file 2) as the basis for inclusion of relevant information. The outcome of interest was the infection of Nigerian ruminants with helminths species of veterinary and zoonotic importance.

A comprehensive literature search was carried out on PubMed, MEDLINE, Google Scholars, AJOL and references of studies that resulted from the search of databases between September, 2016 and March, 2017. To ensure that relevant studies were not omitted, the search was categorized into three stages as broad, narrow and specific search stages. Under the broad search, combinations like helminths of ruminants in Nigeria, prevalence or occurrence of helminths of ruminants in Nigeria were used. Search combinations employed for the narrow search included, but were not limited to, prevalence or occurrence of cestodes, nematodes or trematodes of ruminants in Nigeria. The combinations used under specific search targeted helminth species of ruminants and included, but were not limited to, prevalence or occurrence of *Avitellina* ± *centripunctata*, *Taenia* ± *saginata*/*Cysticercus* ± *bovis*, *Echinococcus*/hydatid ± cyst, *Moniezia* ± *expansa* ± *benedeni*, *Bunostomum* ± *phlebotomum*, *Toxocara* ± *vitulorum*, *Haemonchus* ± *contortus* ± *placei*, *Fasciola* ± *gigantica* ± *hepatica* and *Dicrocoelium* ± *dendriticum* in cattle, sheep and goats. Specific searches were also narrowed to regions and states of the Nigerian federation.

### Inclusion criteria

Studies identified by any of the three search stages were then screened before selection. A study was considered eligible only if: (i) it was carried out in Nigeria, (ii) it was published in English, (iii) it was published between 1970 and 2016, (iv) it was a cross sectional study, (v) the study specified the location in Nigeria where it was conducted, (vi) the sample size and number of positive cases were clearly stated, (vii) the sample size was ≥50, (viii) it reported helminths species of veterinary and zoonotic importance, (ix) the method of diagnosis was stated, (x) parasites were identified at least to the genus level.

In this study, helminths were considered of veterinary importance if they are naturally infective to animals only while those that are naturally infective to man and animals were considered of zoonotic importance. In order to provide data that would guide veterinarians and public health workers in effective diagnosis and treatment as well as policy makers in policy formulation against helminths in Nigeria, endemic helminths were grouped according to their classes as cestodes, nematodes and trematodes.

### Data extraction, collation and analysis

Data pulled out from the eligible studies were: name of author, the year the study was conducted and year it was published, sample size, number of positive cases, state and region of study, study design, type of study, host and helminths species of veterinary and zoonotic importance identified at least to the genus level.

Preliminary analyses including summations, subtractions and divisions were conducted using Microsoft Excel. Statistical and meta-analysis were respectively carried out with Graph-Pad Prism version 4.0 and Comprehensive Meta-Analysis version 3.0. Prevalence for individual studies was determined by multiplying the ratio of cases to sample size by 100. The binomial formula

$$ 95\% CI=\mathrm{p}\pm \mathrm{z}1\hbox{-} \upalpha /2\surd \mathrm{p}\left(1\hbox{-} \mathrm{p}/n\right) $$

was employed to determine the 95% Confidence interval (95% *CI*). It was assumed that the true effect sizes might differ within eligible studies; therefore the random-effects model was used to determine pooled prevalence estimates [35]. Heterogeneity within studies was evaluated using the Cochran’s Q-test while percentage variation in prevalence estimate due to heterogeneity was quantified using the formula *I*^2^ = 100 × (Q-*df*)/Q, where Q is Chi square and *df* is the degree of freedom which is the number of studies minus one. In accordance with the report of Higgins and Thompson, [36], *I*^2^ values of 0, 25, 50 and 75% were considered as no, low, moderate and high heterogeneities, respectively.

## Results

### Bibliographic search and eligible studies

**Fig. 2:**
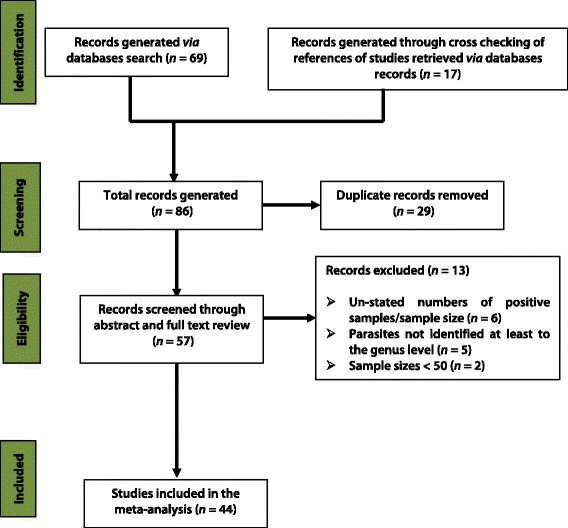
Flow diagram for the selection process of eligible studies

The selection process for eligible studies and the list of excluded studies are presented in Fig. 2 and Additional file 3, respectively. Of the 86 studies retrieved, 69 were from databases while the remaining 17 resulted from checking the lists of references of the studies obtained through the search of databases. Twenty nine duplicate studies were removed after scanning through titles. A total of 13 studies were excluded after detailed abstract and full text review for reasons such as: lack of clearly stated numbers of positive cases/sample sizes (*n* = 6), lack of identification of helminths at least to the genus level (*n* = 5) and sample size less than fifty (*n* = 2). A total of 44 studies were finally included in the meta-analysis.

### Characteristics of eligible studies

The studies analysed were carried out between 1973 and 2016 and published between 1976 and 2016. Ten studies were reported from the North-central, nine from the North-eastern, 11 from the North-western, four each from the South-eastern and South-southern as well as 6 from the South-western regions. A total of 23 937 cases from a sample size of 320 208 were reported. The biological samples collected by the individual studies were blood, faeces and tissues. Thirty two studies reported helminths in cattle, 20 in goats and 12 in sheep. Two of the studies were carried out between 1970 and 1981, three between 1982 and 1993, five between 1994 and 2005 as well as 34 between 2006 and 2016. Twenty eight studies had sample sizes ≤1000, 5 had sample sizes between 1001 and 2000 and 11 had sample sizes greater than 2000. Thirty six, six, and two studies were abattoir-based, farm-based and market-based, respectively, while ten, 31 and three of the studies were diagnosed using macroscopy, microscopy and serology respectively (Table 1).

**Table 1:** List and characteristics of the 44 eligible studies

Year of study	Region	Host	Type of study	Method of diagnosis	Sample size	Cases	Prev. (%)	Study RN
2002	North-east	G/S	Farm-based	Microscopy	249	126	50.60	[16]
2007	North-west	C/G/S	Abattoir-based	Microscopy	300	100	33.33	[17]
2012/2013	North-central	C/G/S	Farm-based	Microscopy	326	298	91.41	[18]
2013/2014	North-central	C/G/S	Abattoir-based	Microscopy	2508	642	25.60	[19]
2016	North-east	C	Abattoir-based	Microscopy	208	187	89.90	[22]
2008	North-west	C	Abattoir-based	Macroscopy	11 804	315	2.67	[23]
2013	North-west	C	Abattoir-based	Serology	285	69	24.21	[24]
2013	South-west	C	Abattoir-based	Microscopy	397	163	41.06	[25]
2013	South-east	G	Abattoir-based	Microscopy	200	185	92.50	[26]
2013	North-central	G	Abattoir-based	Microscopy	248	183	73.79	[27]
2003/2004	North-west	C/G/S	Abattoir-based	Microscopy	76 702	61	0.08	[28]
2011	South-west	C/G/S	Farm-based	Microscopy	1171	251	21.43	[68]
2012/2013	South-south	C	Abattoir-based	Macroscopy	22 259	382	1.72	[69]
2012	North-west	C	Abattoir-based	Serology	386	66	17.10	[70]
2012	North-east	C	Abattoir-based	Macroscopy	3015	657	21.79	[71]
1973/1974	North-east	C	Abattoir-based	Macroscopy	14 270	4524	31.70	[72]
2012/2013	North-central	G/S	Market-based	Microscopy	1002	552	55.09	[73]
2009/2010	South-south	C	Abattoir-based	Microscopy	251	156	62.15	[74]
2013	North-central	C	Farm-based	Serology	686	536	78.13	[75]
2011/2012	North-west	G/S	Abattoir-based	Microscopy	300	242	80.67	[76]
2015	South-south	C	Abattoir-based	Microscopy	514	35	6.81	[77]
2005	South-west	C	Abattoir-based	Macroscopy	483	75	15.53	[78]
2010–2013	North-east	C	Abattoir-based	Macroscopy	6007	288	4.79	[79]
2016	North-east	C	Abattoir-based	Microscopy	208	62	29.81	[80]
2013	North-west	C	Abattoir-based	Microscopy	224	62	27.68	[81]
2009	North-west	C	Abattoir-based	Microscopy	200	30	15.00	[82]
1986	North-west	C	Abattoir-based	Microscopy	502	156	31.08	[83]
2011	North-west	C	Farm-based	Microscopy	1525	820	53.77	[84]
2009	South-east	C/G	Abattoir-based	Microscopy	1138	525	46.13	[85]
1991/1992	South-west	G	Abattoir-based	Microscopy	1080	896	82.96	[86]
2013	North-central	G	Abattoir-based	Microscopy	248	183	73.79	[87]
2013/2014	South-west	C/G/S	Farm-based	Microscopy	170	132	77.65	[88]
2010	North-west	C	Abattoir-based	Macroscopy	285	5	1.75	[89]
2014	South-west	G	Market-based	Microscopy	400	303	75.75	[90]
1985	South-east	C	Abattoir-based	Macroscopy	942	38	4.03	[91]
1999–2002	South-east	C	Abattoir-based	Macroscopy	25 800	6750	26.16	[92]
2010/2011	South-south	G	Abattoir-based	Microscopy	213	161	75.59	[93]
2015	North-central	C	Abattoir-based	Microscopy	160	55	34.38	[94]
1997–1999	North-central	C	Abattoir-based	Microscopy	14 372	1924	13.39	[95]
1973–1975	North-east	C/G/S	Abattoir-based	Microscopy	3322	1202	36.18	[96]
2012	North-east	C	Abattoir-based	Microscopy	350	122	34.86	[97]
2010	North-central	G/S	Abattoir-based	Microscopy	110	59	53.64	[98]
2006	North-east	G/S	Abattoir-based	Macroscopy	124 888	78	0.06	[99]
2011	North-central	C	Abattoir-based	Microscopy	500	281	56.20	[100]

C: Cattle; G: Goats; S: Sheep; Prev.: Prevalence; RN: Reference number

### Regional distribution of eligible studies

The studies were distributed across 19 Nigerian States. Studies were concentrated mostly in the North-western region 11 (25.0%) and Sokoto State five (11.4%), followed by the North-central region 10 (22.7%) as well as Oyo and Rivers States four (9.1%). The least number of studies were reported in the South-southern region, four (9.19%) as well as Adamawa, Imo and Niger States, one (2.3%) as presented in Fig. 1.

### Pooled prevalence estimate and heterogeneity analysis

The overall pooled prevalence estimate (PPE), PPEs for different strata and heterogeneities are presented in Table 2. Individual prevalence of eligible studies ranged between 0.06 and 92.50%. The study revealed an overall pooled prevalence estimate of 7.48% (95% *CI*: 7.38–7.57) from 23 937 cases and 320 208 ruminants. Regional pooled prevalence estimates ranged between 2.08% (95% *CI*: 1.99–2.18) in the North-western region and 49.18% (95% *CI*: 47.55–50.80) in the South-western region. PPEs among different host species ranged between 1.90% (95% *CI*: 1.78–2.02) and 12.55% (95% *CI*: 12.39–12.72). Based on the period of study, prevalence estimates ranged between 4.49% (95% *CI*: 4.39–4.58) among studies published between 2006 and 2016 and 43.19% (95% *CI*: 41.24–45.14) for studies published between 1982 and 1993. Pooled prevalence estimates in relation to sample sizes ranged between 5.52% (95% *CI*: 5.44–5.60) for studies with sample sizes greater than 2000 and 51.45% (95% *CI*: 50.17–52.73) for studies with sample sizes between 1001 and 2000. Prevalence estimates in relation to study settings ranged between 6.65% (95% *CI*: 6.56–6.73) for abattoir-based and 60.98% (95% *CI*: 58.37–63.55) for market-based studies. PPEs in relation to methods of diagnosis ranged between 6.25% (95% *CI*: 6.15–6.36) for studies diagnosed using macroscopy and 49.45% (95% *CI*: 46.75–52.14) for studies diagnosed using serology. The PPEs for helminths of zoonotic importance in Nigerian ruminants were 0.11% (95% *CI*: 0.09–0.12), 13.60% (95% *CI*: 12.46–14.80), 13.84% (95% *CI*: 13.55–14.13) and 15.81% (95% *CI*: 15.51–16.11) for *Echinococcus*/hydatid cysts, *Oesophagostomum* species, *Fasciola gigantica* and *T. saginata*/*Cysticercus bovis* respectively (Table 2).

**Table 2:** Pooled prevalence estimates of helminths in Nigerian ruminants based on different strata

Variables	No. of Studies	Pooled prevalence estimates			(95% *CI*)	Heterogeneity	
		Sample size	Cases	Prev. (%)		*I*^*2*^ (%)	Q-*P*
Region							
North-central	10	20 160	4713	23.38	22.80–23.97	99.71	0.000
North-east	9	152 517	7246	4.75	4.64–4.86	99.84	0.000
North-west	11	92 513	1926	2.08	1.99–2.18	99.79	0.000
South-east	4	28 080	7498	26.70	26.19–27.22	99.46	0.000
South-south	4	23 237	734	3.16	2.94–3.39	99.83	0.000
South-west	6	3701	1820	49.18	47.55–50.80	99.52	0.000
Hosts							
Cattle	32	154 953	19 446	12.55	12.39–12.72	99.75	0.000
Goat	20	113 563	3510	3.09	2.99–3.19	99.60	0.000
Sheep	12	51 692	981	1.90	1.78–2.02	99.55	0.000
Study period							
1970–1981	2	17 592	5726	32.55	31.86–33.25	95.93	0.000
1982–1993	3	2524	1090	43.19	41.24–45.14	99.75	0.000
1994–2005	5	117 606	8936	7.60	7.45–7.75	99.87	0.000
2006–2016	34	182 486	8185	4.49	4.39–4.58	99.76	0.000
Sample size							
≤1000	28	9345	4070	43.55	42.54–44.57	98.81	0.000
1001–2000	5	5916	3044	51.45	50.17–52.73	99.46	0.000
> 2000	11	304 947	16 823	5.52	5.44–5.60	99.92	0.000
Study type							
Abattoir-based	36	314 679	20 919	6.65	6.56–6.73	99.79	0.000
Farm-based	6	4127	2163	52.41	50.87–53.94	99.76	0.000
Market-based	2	1402	855	60.98	58.37–63.55	97.98	0.000
MOD							
Macroscopy	10	209 753	13 112	6.25	6.15–6.36	99.91	0.000
Microscopy	31	109 098	10 154	9.31	9.14–9.48	99.63	0.000
Serology	3	1357	671	49.45	46.75–52.14	99.49	0.000
Overall	44	320 208	23 937	7.48	7.38–7.57	99.78	0.000

*P* < 0.001 for all strata; *CI*: Confidence interval; *I*^2^: Inverse variance index; MOD: Method of diagnosis; Prev.: Prevalence; Q-*P*: Cochran’s *P*-value

**Fig. 3:**
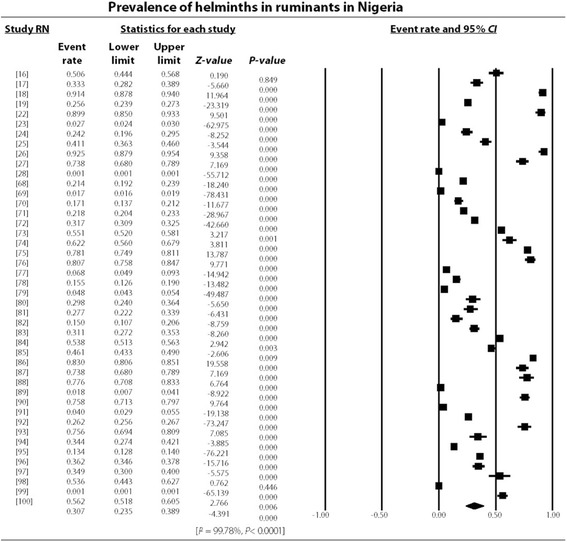
Forest plot for the prevalence of helminths of veterinary and zoonotic importance in Nigerian ruminants. RN: Reference number

**Fig. 4:**
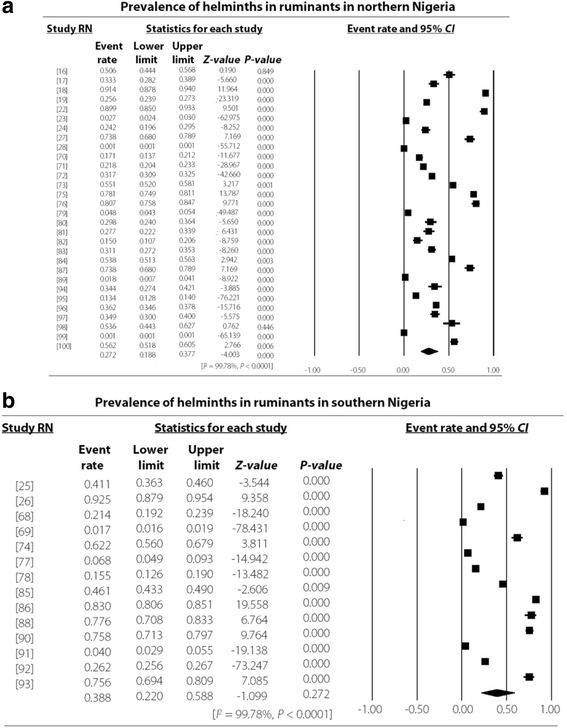
Forest plot for the prevalence of helminths of veterinary and zoonotic importance in ruminants in Northern and Southern Nigeria. RN: Reference number

**Fig. 5:**
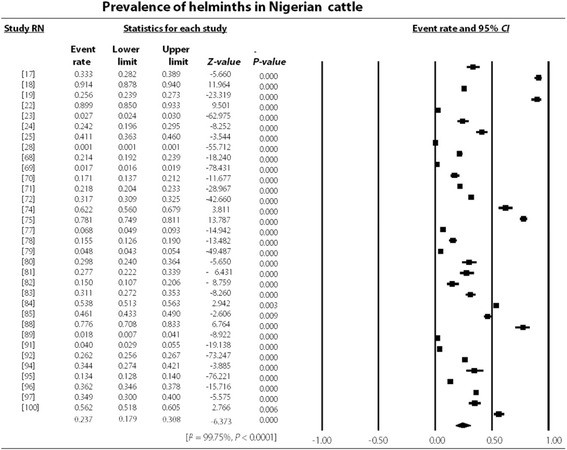
Forest plot for the prevalence of helminths of veterinary and zoonotic importance in Nigerian cattle. RN: Reference number

**Fig. 6:**
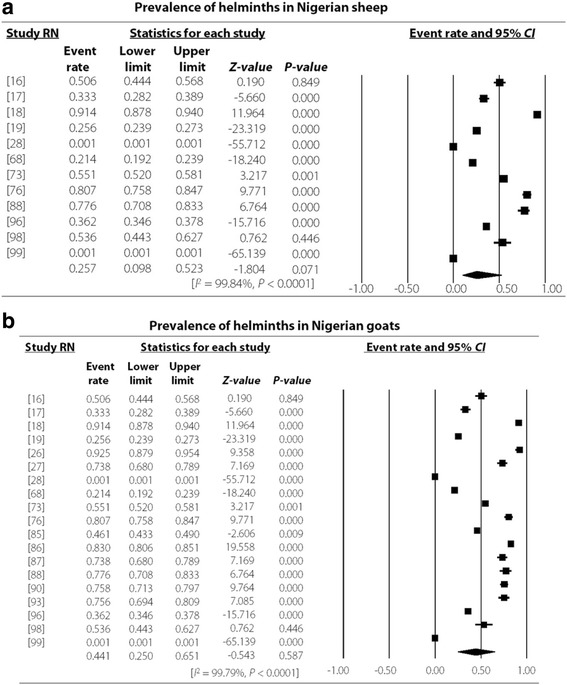
Forest plot for the prevalence of helminths of veterinary and zoonotic importance in goats and sheep in Nigeria. RN: Reference number

The study revealed an overall high degree of heterogeneity (99.78%, 95% *CI*: 7.38–7.57, *P* < 0.001) which persisted even in different strata such as the Northern (99.78%, 95% *CI*: 5.15–5.32, *P* < 0.001) and Southern regions (99.78, 95% *CI*: 17.95–18.60, *P* < 0.001) as well as hosts like cattle (99.75, 95% *CI*: 12.39–12.72, *P* < 0.001), sheep (99.55%, 95% *CI*: 1.78–2.02, *P* < 0.001) and goats (99.60%, 95% *CI*: 2.99–3.19, *P* < 0.001) as presented in Figs. 3, 4, 5, and 6. Most (48.15%) of the parasites reported in ruminants were nematodes. The most prevalent species of cestode, nematode and trematode were *T. saginata*/*Cysticercus bovis* (15.81%), *Strongyloides papillosus* (32.06%) and *Paramphistomum* spp. (15.51%) while *Taenia* spp., *Strongyloides papillosus* and *Fasciola gigantica* respectively had the widest geographical distribution (Table 3).

**Table 3:** Pooled prevalence estimates and distribution of helminths species according to class of parasites

Parasites	Number of studies	Pooled prevalence estimates			(95% *CI*)	Heterogeneity	
		Sample size	Cases	Prev. (%)		*I*^*2*^ (%)	Q-*P*
HVI Cestodes							
*Moniezia expansa*	9	5785	368	6.36	5.75–7.02	99.51	0.000
*Avitellina centripunctata*	3	712	33	4.63	3.21–6.45	95.01	0.000
*Taenia* spp.	10	3224	104	3.23	2.64–3.90	99.17	0.000
*Moniezia benedeni*	4	4643	77	1.66	1.31–2.07	97.62	0.000
Overall (Cestodes)	–	14 364	582	4.05	3.73–4.39	97.82	0.000
Nematodes							
*Strongyloides papillosus*	13	7671	2459	32.06	31.01–33.11	99.90	0.000
*Gongylonema* spp.	1	248	41	16.53	12.13–21.75	0.00	0.594
*Gaigeria* spp.	1	248	35	14.11	10.03–19.08	0.00	0.742
*Bunostomum phlebotomum*	9	4513	407	9.02	8.20–9.89	94.16	0.000
*Trichostrongylus* spp.	7	3300	287	8.70	7.76–9.71	99.01	0.000
*Ostertagia* spp.	5	1160	99	8.53	6.99–10.29	0.00	0.436
*Trichuris globulosa*	8	4930	337	6.84	6.15–7.58	87.56	0.005
*Cooperia pectinata*	2	2663	173	6.50	5.59–7.50	0.00	0.335
*Haemonchus contortus*	8	4104	234	5.70	5.01–6.46	98.78	0.000
*Chabertia ovina*	2	464	25	5.39	3.52–7.85	0.00	0.844
*Nematodirus* spp.	4	1905	87	4.57	3.67–5.60	89.94	0.002
*Toxocara vitulorum*	10	6634	283	4.27	3.79–4.78	98.65	0.000
*Trichuris ovis*	7	4591	71	1.55	1.21–1.95	87.93	0.004
Overall (Nematodes)	–	42 431	4538	10.70	10.40–10.99	99.67	0.000
Trematodes							
*Paramphistomum* spp.	12	9180	1424	15.51	14.78–16.27	91.49	0.001
*Eurytrema pancreaticum*	3	672	81	12.05	9.69–14.76	98.76	0.000
*Schistosoma bovis*	6	1926	191	9.92	8.62–11.34	95.54	0.000
*Dicrocoelium hospes*	5	7809	641	8.21	7.61–8.84	98.57	0.000
*Dicrocoelium dendriticum*	4	3436	152	4.42	3.76–5.17	71.93	0.059
*Gastrothylax* spp.	2	464	14	3.02	1.66–5.01	46.71	0.171
Overall (Trematodes)	–	23 478	2503	10.66	10.27–11.06	98.79	0.000
HZI							
*T. saginata*/*C. bovis*	5	58 925	9315	15.81	15.51–16.11	98.79	0.000
*Fasciola gigantica*	20	53 402	7390	13.84	13.55–14.13	99.97	0.000
*Oesophagostomum* spp.	7	3397	462	13.60	12.46–14.80	0.00	0.799
*Echinococcus*/Hydatid cyst	4	202 160	213	0.11	0.09–0.12	77.86	0.034
Overall (HZI)		317 884	17 380	5.47	5.39–5.55	99.95	0.000

*CI*: Confidence interval; HVI: Helminths of veterinary importance; HZI: Helminths of zoonotic importance; *I*^*2*^: Inverse variance index; Prev.: Prevalence; Q-*P*: Cochran’s *P*-value

## Discussion

Several studies have documented individual divisional, provincial and regional reports on helminth parasites of ruminants in different parts of Nigeria. However, information on the national prevalence of these parasites is lacking. The evidence available shows that this is probably the first meta-analysis to consider endemic helminths of ruminants, their prevalence and distribution across Nigeria. The study was necessary to provide useful epidemiological information required for the institution of control programmes that will help in reducing economic losses and public health problems associated with these helminths.

The overall pooled prevalence estimate of 7.48% observed in Nigerian ruminants is lower than reports of other meta-analysis from Ethiopia [37, 38]. The variations in these PPEs may be attributable to factors including grazing habits, nutritional status, husbandry and production systems, host immunological status [39], availability of intermediate hosts as well as the number of viable infective larvae and eggs in the environment [40]. The differences between time of sample collection and analysis as well as the specificity and sensitivity of the diagnostic methods employed by the various studies may also be possible reasons for the variations in the PPEs. Studies from Ethiopia [41] and Andhra Pradesh, India [42] also reported similar helminth species as those reported in Nigeria during the period under review.

Pooled prevalence estimates in relation to regions was highest in South-western Nigeria probably due to the forested nature, the longer periods of rainfall, lower temperatures, lower humidity and high soil moisture in the region [33]. Failure of control programmes such as adequate sanitation, control of intermediate hosts and strategic deworming due to inadequate funding may also account for the higher prevalence in south-west Nigeria. Majority of Nigerian livestock are raised in Northern Nigeria explaining the higher number of studies reported in the region.

Yearly distribution of studies shows that most of the studies were published between 2006 and 2016 suggesting an increase in research during the last few years out of the four decades reviewed. This may be due to increased awareness and research on animal health. The study revealed a drastic decline in the pooled prevalence of similar helminths from 47.76% during 1982 and 1993 to 4.49% during 2006 and 2016. With the increased specificity and sensitivity of current diagnostic techniques, the recent decline in the prevalence of helminths suggests increased awareness of the socio-economic and public health consequences of these infections by farmers, improvements in quality of veterinary services, management practices as well as improved hygiene and sanitation levels.

Studies with larger sample sizes give more representation of the study population, and are believed to provide more reliable findings. Despite these advantages, over 64% of the studies had sample sizes of 1000 and less. The smaller sample sizes may not be unconnected with the lack of grants for supporting research in Nigeria. The higher prevalence observed in cattle as compared to sheep and goats may be due to factors related to differences in host’s susceptibility, genetic make-up, defence mechanisms and parasite host specificity. The open grazing of cattle in areas used by humans for defecation as opposed the raising of sheep and goats in backyard housing may explain the higher prevalence reported in cattle.

From the economic standpoint, cattle and small ruminants (sheep and goats), serve as major sources of income and livelihood, and contribute 50 and 35% of the total meat produced in Nigeria, respectively [25, 32] despite the fact that over 90% of them are managed traditionally with inadequate veterinary care [43]. Therefore, this study which provides information on the burden of helminth infections in cattle and small ruminants became necessary to curtail economic losses that may be associated with unidentified and uncontrolled helminth infections.

Majority of the reported nematode species are parasites of cattle, sheep and goats with the exception of *Toxocara vitulorum* and *Trichostrongylus* spp., which are mainly parasites of cattle. Nematodes like *Oesophagostomum* spp. may also be of public health concern. The presence of *Haemonchus contortus* is of particular concern due to its high pathogenecity and economic importance in sheep and goats [44].

The distribution of studies in relation to study settings shows that majority (82.2%) of the studies were abattoir-based, probably due to the ease of collecting samples from slaughtered animals, especially with the challenges of on-farm studies such as unwillingness of herdsmen to allow researchers access to their animals, problems of restraining large animals like cattle and insecurity in the rural areas where most of the livestock is raised. The epidemiological significance of the highest PPE reported in livestock markets is the risk of initiating new endemic foci for these infections especially those of public health concerns like cysticercosis and cystic echinococcosis. The presence of zoonotic helminths in food animals slaughtered for human consumption during this period is of public health concern. To ensure food safety, quality veterinary meat inspection is suggested to curtail the transmission of these helminths to humans.

*Cysticercus bovis* was the most prevalent (15.81%) of the five species of cestodes reported in Nigeria. This PPE is considerably higher than the ranges documented in other developing countries of Africa (0.2–5.6%) [45–47] and elsewhere (0.09–3.0%) [48–50]. The occurrence of this metacestode alongside hydatid cysts in food animals that entered the food chain is a threat to public health considering their association respectively with cysticercosis and cystic echinococcosis in humans.

*Paramphistomum* spp. was the most prevalent of all the species of trematodes reported in Nigeria. This may be due to the massive asexual multiplication of helminths of the genus *Paramphistomum* in snail intermediate hosts and the long lifespan of these helminths that usually results in a constant source of infection for successive generations of snails [51]. Substantive evidence shows that various genera of these snails (*Archachatina*, *Limicolaria* and *Oncomelania*) are endemic in Nigeria [52–55]. These reports have justified the occurrence of different trematodes like *Fasciola gigantica, Dicrocoelium* spp., *Eurytrema pancreaticum* and *Schistosoma bovis* among others across Nigeria. Though there are no documented reports of human infections with zoonotic flukes like *Fasciola gigantica, Dicrocoelium dendriticum* and *Eurytrema pancreaticum* in Nigeria, reports elsewhere [56–59] showed that they may be potential threats to public health in Nigeria. This suggests the need for studies in humans to determine the status of these parasites in Nigerians.

The grouping of helminths according to their phylogenetic classes was based on the fact that members of these groups share common control measures. For instance, while nematodes of ruminants have direct life cycle and are pasture-borne, cestodes and trematodes that have indirect life cycles are arthropod-borne and gastropod-borne parasites, respectively [3, 60]. Consequently, while nematode control targets rotational grazing of ruminants, the control of cestodes and trematodes usually focus on reducing the numbers of arthropod and gastropods intermediate hosts in the environment. In addition, majority of anthelmintics used for chemotherapeutic control are also classified as anticestodals (e.g. praziquantel, nitroscanate), antinematodals (e.g. piperazine, tetrahydropyrimidines) and antitrematodals (e.g. benzimidazoles, salicylanides) for these phylogenetic classes [61–63].

Three diagnostic methods (macroscopy, microscopy and serology) were employed by the 44 studies analyzed. Macroscopy was used basically for the gross identification of cystic conditions caused by larval stages of cestodes like *C. bovis* and hydatid cyst as well as adult helminths like *Fasciola* spp. Microscopy was used for the identification of helminth eggs while serology was used for antigen/antibody detection. Though all these techniques are valuable in the diagnosis of helminth infections, they are not without limitations. While macroscopy may easily miss non-prominent lesions during gross examination of tissues, sample preparation for microscopy may be time-consuming, labour intensive and requires expertise. Serology which is the most sensitive and specific of these three diagnostic techniques is associated with false positive results and is limited to the detection of only few helminth species. These limitations may contribute to the low prevalence observed and the inability of some of the studies to identify helminths to the species level. Due to these deficiencies, highly sensitive and specific molecular techniques earlier described [64–67] may provide a better understanding of the status of helminths parasites in Nigeria.

The findings of this study have several implications. Looking from the epidemiological point of view, the detection of helminths in congregations of livestock like markets may suggest possible increased risk of transmission of these parasites as majority of farmers buy these animals from the markets and introduce them into their herds without any veterinary care. On the other hand, the presence of these helminths in ruminants on farms may probably cause contamination of grazing pasture and sources of drinking water for these animals resulting in new foci of infections. There are obvious public health implications of finding zoonotic helminths like hydatid cyst, *Cysticercus bovis*, *Fasciola gigantica* and *Oesophagostomum* spp. among others in ruminants. These include the risk of environmental contamination that may result in human infections or of acquiring such infections through the consumption of slaughtered food animals that enter the food chain. These parasites are associated with different conditions in humans ranging from diarrhoea, retarded growth, intellectual and cognitive retardation [29] to cystic echinococcosis and cysticercosis.

### Limitations

Though this study provided useful epidemiological information on the prevalence and distribution of endemic helminths in Nigeria, which will be useful in disease control, it is not devoid of limitations. First, there were uneven distributions of studies across states, regions, hosts, study period, study types and sample sizes, This implies that the findings may not accurately represent the situation for Nigeria. Another setback is the fact that despite the distribution of eligible studies across the six Nigerian regions, studies were published from only nineteen of the thirty-six states.

## Conclusions

Helminths of veterinary and medical importance are prevalent in Nigeria with overall PPE of 7.48%. There was a 43.3% decline in the pooled prevalence of helminths over a period of 13 years. The highest pooled prevalence estimates were observed in the South-western region and among cattle. *Strongyloides papillosus* was the most prevalent of all the helminths while *Fasciola gigantica* had the widest geographical distribution across Nigeria. High degrees of heterogeneity were observed within studies and different strata. On-farm good agricultural practices including effective strategic deworming of livestock according to parasites’ seasonality and abundance, ranching instead of nomadism, standard veterinary meat inspection and adequate hygiene and sanitation in abattoirs and livestock markets will reduce the economic, public health and veterinary threats caused by these parasites.

## Additional files

Additional file 1: Multilingual abstracts in the five official working languages of the United Nations. (PDF 251 kb)

Additional file 2: Preferred Reporting Items for Systematic Review and Meta-Analysis (PRISMA) checklist. (DOC 62 kb)

Additional file 3: List of studies excluded from the meta-analysis. (DOCX 19 kb)

## Abbreviations

AJOL: African Journals OnLine
C: Cattle
*CI*: Confidence interval
FCT: Federal Capital Territory
G: Goat
HVI: Helminths of veterinary importance
HZI: Helminths of zoonotic importance
*I*^2^: Inverse variance index
MOD: Method of diagnosis
PPE(s): Pooled prevalence estimate(s)
Prev: Prevalence
PRISMA: Preferred Reporting Items for Systematic Review and Meta-Analysis
Q: Cochran’s heterogeneity statistic
Q-*P*: Cochran’s *P*-value
RN: Reference number
S: Sheep
