# Supplementary material for: Helminths of veterinary and zoonotic importance in Nigerian ruminants: a 46-year meta-analysis (1970–2016) of their prevalence and distribution
Source: Infect Dis Poverty. 2018 May 29;7:52. doi: 10.1186/s40249-018-0438-z (PMC5972411; doi:10.1186/s40249-018-0438-z)
Supplement: Supplementary file 1 — Multilingual abstracts in the five official working languages of the United Nations. (PDF 251 kb) [file 40249_2018_438_MOESM1_ESM.pdf]

Translation of the abstract into the five official working languages of the United Nations

الديدان الطفيلية ذات الأهمية البيطرية والحيوانية في الحيوانات المجترة بنيجيريا: تحليل تلوي لمدة 46 سنة (1970-2016) لانتشارها وتوزيعها

سولومون نغوتور كارشيما ، بيتي فيف مايكاي ، جاكوب كوادا باغي كواجا

الملخص

الخلفية : تلعب صناعة الثروة الحيوانية دوراً حيوياً في اقتصاد نيجيريا. وهي بمثابة مصدر رئيسي للدخل والمعيشة لغالبية النيجيريين من المستوطنين الريفيين الذين يساهمون بنحو 5.2 ٪ في إجمالي الناتج المحلي القومي. إلا أن الديدان الطفيلية تسبب خسائر اقتصادية بسبب النقص في إنتاج الحليب وفي زيادة الوزن والخصوبة وجودة الذبيحة. تسبب الديدان الطفيلية حيوانية المنشأ لأصول الثروة الحيوانية مشاكل صحية عند البشر.

الطرق : باستخدام معايير الإبلاغ المفضلة للمبادئ التوجيهية للمراجعة المنهجية والتحليل التلوي (PRISMA) ، تم تحديد مدى انتشار وتوزيع الديدان الطفيلية ذات الأهمية البيطرية والحيوانية في الحيوانات المجترة النيجيرية في تحليل تلوي للبيانات المنشورة بين أعوام 1970 و 2016. كانت البيانات موزعة على أساس المناطق ، والمضيف ، وفترات الدراسة ، وحجم العينات وأنواع الدراسة في حين تم تصنيف الديدان الطفيلية إلى سلالات الديدان الشريطية، والديدان الخيطية والمثقوبات.

النتائج : أظهرت البيانات من 44 دراسة تم الإبلاغ عنها في 19 ولاية نيجيرية تقديرًا شاملًا لمدى التفشي (PPE) بنسبة 7.48٪ (مجال الموثوقية 95٪: 7.38-7.57) للديدان الطفيلية ذات الأهمية البيطرية والحيوانية من إجمالي 320،208 من الحيوانات المجترة. لاحظنا تباينًا كبيرًا (P <0.001) بين نطاق تقدير تفشي الانتشار الشامل بنسبة 1.90٪ (مجال الموثوقية 95٪: 1.78-2.02) و 60.98٪ (مجال الموثوقية 95٪: 58.37-63.55) تم إثباته عبر طبقات مختلفة. وقد لوحظ عدم تجانس عالٍ (مجال الموثوقية 99.78%, 95%: 7.38-7.57). كانت الديدان الأسطوانية الحليمية هي الأكثر انتشارًا (السابق: 32.02٪ ، 95٪ CI: 31.01-33.11) ، بينما كان لدى المتورقات العملاقة أوسع توزيع جغرافي.

الخلاصة : تنتشر الديدان الطفيلية ذات الأهمية البيطرية والحيوانية في الحيوانات المجترة وموجودة بكثرة في أنحاء نيجيريا. تُظْهِر النتائج التي توصلنا إليها أن الديدان الطفيلية للحيوانات المجترة قد تكون أيضًا أسبابًا محتملة للمرض في الإنسان وللخسائر الاقتصادية في صناعة الثروة الحيوانية في نيجيريا. وقد لوحظ عدم التجانس العالي داخل الدراسات والطبقات المختلفة. يجب تنفيذ الممارسات الزراعية الجيدة في المزارع ، والتفتيش البيطري المعياري للحوم والنظافة الملائمة والصرف الصحي في المجازر والمزارع وأسواق الماشية في نيجيريا، من أجل الحد من التهديدات الاقتصادية والصحة العامة والبيطرية بسبب هذه الديدان الطفيلية.

Translated from English version into Arabic by Free Bird and Ali Mohammed, through

TRANSLATORS
WITHOUT BORDERS

尼日利亚反刍动物中重要兽类和人兽共患蠕虫的疾病流行率和分布情况：一项 46 年（1970-2016）荟萃分析

Solomon Ngutor Karshima, Beatty-Viv Maikai, Jacob Kwada Paghi Kwaga

摘要

引言：畜牧业在尼日利亚的经济发展中发挥着至关重要的作用。它是大多数尼日利亚农民的主要收入和生计来源，占全国国内生产总值（GDP）的 5.2%。然而，蠕虫造成牛奶产量减

少，体重增加，肥力和胴体品质下降，进而导致经济损失。家畜源性人兽共患蠕虫病也会影响人类健康。
方法：本研究按照系统评估首选报告项目和荟萃分析（PRISMA）指南，对尼日利亚 1970 年至 2016 年间公布的数据进行荟萃分析，确定了尼日利亚反刍动物中主要兽类和人兽共患蠕虫的流行率和分布情况。我们根据地区、宿主、研究周期、样本量和研究类型对数据进行分层；蠕虫在系统发育上分为绦虫、线虫和吸虫。
结果：19 个尼日利亚国家的 44 项研究数据显示，320,208 只反刍动物的重要兽类和人兽共患传染蠕虫病总体汇集患病率（PPE）约为 7.48％（95％ *CI*：7.38–7.57）。我们观察到不同层级报告的 PPEs 的差异显著（*P* < 0.001）：1.90％(95％ *CI*：1.78–2.02）– 60.98％（95％ *CI*：58.37–63.55）。并观察到高度异质性(99.78%, 95% *CI*: 7.38-7.57)。乳突类圆线虫（*Strongyloides papillosus*）最常见 (流行率: 32.02%, 95% *CI*: 31.01-33.11), 而 *Fasciola gigantica* 地理分布最广。
结论：在兽医和人兽共患领域具有重要意义的蠕虫在尼日利亚各地的反刍动物中普遍存在。研究结果表明，反刍动物体内蠕虫可能是导致人类发病，并造成尼日利亚畜牧业的经济损失的原因。这在不同研究和不同层级中呈高度异质性。尼日利亚的农场、屠宰场和畜牧市场需要实施良好的农业规范、标准的肉类检验，以及充分的清洁卫生措施，以降低这些蠕虫对当地经济、公共卫生和兽医方面的威胁。

Translated from English version into Chinese by Jin Chen, edited by Pin Yang

CHINESE CENTER FOR DISEASE CONTROL AND PREVENTION
NATIONAL INSTITUTE OF PARASITIC DISEASES

Les helminthes d'importance vétérinaire et zoonotique chez les ruminants nigérians : une méta-analyse de leur prévalence et de leur répartition sur une période de 46 ans (1970-2016)

Solomon Ngutor Karshima, Beatty-Viv Maikai, Jacob Kwada Paghi Kwaga

Résumé
Contexte : L'industrie du bétail joue un rôle essentiel dans l'économie du Nigéria. Elle constitue une source importante de revenus et de moyens de subsistance pour la majorité des nigérians qui vivent dans des communautés rurales et elle représente environ 5,2 % du PIB national. Cependant, les helminthes entraînent des pertes économiques en raison des diminutions de la production de lait, de la prise de poids, de la fertilité et de la qualité de la carcasse. Les helminthes zoonotiques qui proviennent du bétail provoquent des problèmes de santé qui affectent les humains.
Méthodes : Les lignes directrices pour l'écriture et la lecture des revues systématiques et des méta-analyses (PRISMA), ont permis de déterminer la prévalence et la distribution des helminthes d'importance zoonotique et vétérinaire chez les ruminants nigérians dans une méta-analyse des données publiées entre 1970 et 2016. Les données ont été stratifiées en fonction des régions, des hôtes, des périodes d'étude, des tailles des échantillons et des types d'études tandis que les helminthes ont été phylogénétiquement regroupés en cestodes, nématodes et trématodes.
Résultats : Les données de 44 études effectuées à travers 19 états nigérians ont révélé un taux de prévalence cumulée (TPC) global de 7,48 % (95 % IC : 7,38-7,57) pour les helminthes d'importance

vétérinaire et zoonotique sur un total de 320208 ruminants. Nous avons observé un écart important (p < 0,001) entre l'étendue des TPC de 1,90 % (95 % IC : 1,78-2,02) et 60,98 % (95 % IC : 58,37-63,55) signalé dans différentes strates. Nous avons remarqué une forte hétérogénéité (99,78 %, 95 % IC : 7,38-7,57). *Strongyloides papillosus* était le plus prévalent (Prév : 32,02 %, 95 % IC : 31,01-33,11), tandis que *Fasciola gigantica* était le plus largement réparti géographiquement.

Conclusion : Les helminthes d'importance vétérinaire et zoonotique sont prévalent chez les ruminants et bien répartis à travers le Nigéria. Nos résultats montrent que les helminthes des ruminants pourraient également être des causes de morbidité chez les humains et de pertes économiques dans l'industrie du bétail au Nigéria. Nous avons observé une forte hétérogénéité dans les études et dans les diverses strates. Des pratiques agricoles efficaces sur les exploitations, une inspection vétérinaire normalisée de la viande ainsi qu'une hygiène et un assainissement adéquats dans les abattoirs, les fermes et sur les marchés de vente de bétail doivent être mis en place au Nigéria afin de réduire les menaces économiques, vétérinaires et de santé publique causées par ces helminthes.

Translated from English version into French by Crystelle 14 and Aurore Dary, through

TRANSLATORS
WITHOUT BORDERS

Гельминты, имеющие ветеринарное и зоонозное значение, у нигерийских жвачных животных: метаанализ данных об их распространенности и распределении за 46 лет (1970–2016 гг.)

Соломон Нгутор Каршима (Solomon Ngutor Karshima), Битти-Вив Майкай (Beatty-Viv Maikai), Джейкоб Квада (Jacob Kwada)

Выдержка

Предыстория: Животноводство играет жизненно важную роль в экономике Нигерии. Оно служит важным источником дохода и средств к существованию для большинства нигерийцев, живущих в сельской местности, и составляет около 5,2% национального валового внутреннего продукта (ВВП). Однако гельминты вызывают экономические потери из-за сокращения производства молока, уменьшения прироста веса, снижения плодовитости и качества туши. Зоонозные гельминты у домашних животных вызывают проблемы со здоровьем у людей.

Методы: В соответствии с Предпочтительными параметрами отчетности для систематических обзоров и метаанализов (PRISMA) распространенность и распределение гельминтов, имеющих ветеринарное и зоонозное значение, у нигерийских жвачных животных были определены в метаанализе данных, опубликованных в период с 1970 по 2016 год. Данные были стратифицированы по следующим факторам: регион, организм-хозяин, период исследования, размер выборки и тип исследования; при этом гельминты были разбиты на следующие филогенетические группы: цестоды, нематоды и трематоды.

Результаты: Данные, полученные из 44 исследований, проведенных в 19 нигерийских штатах, показали общую оценку распространенности (ООР) на уровне 7,48% (95% ДИ: 7,38–7,57) для гельминтов, имеющих ветеринарное и зоонозное значение, в целом у 320 208 жвачных животных. Мы наблюдали значительное различие (p <0,001) между диапазонами ООР на уровне 1,90% (95% ДИ: 1,78–2,02) и 60,98% (95% ДИ: 58,37–63,55), зарегистрированными в разных стратах. Наблюдалась высокая гетерогенность (99,78%, 95% ДИ: 7,38–7,57). *Strongyloides papillosus* был наиболее преобладающим видом (Распр.: 32,02%, 95% ДИ: 31,01—33,11), в то время как у вида *Fasciola gigantica* было наиболее широкое географическое распределение.
Вывод: Гельминты, имеющие ветеринарное и зоонозное значение, распространены среди нигерийских жвачных животных и широко распределены по всей Нигерии. Наши результаты показывают, что гельминты у жвачных животных также могут быть причинами заболеваемости у людей и экономических потерь в животноводстве Нигерии. Значительные различия наблюдались среди исследований и в различных стратах. В Нигерии должны быть внедрены надлежащие методы ведения сельского хозяйства на фермах, стандартная ветеринарная проверка мяса и надлежащие санитарно-гигиенические меры на скотобойнях, фермах и животноводческих рынках, чтобы уменьшить экономические, общественные и ветеринарные угрозы, вызванные этими гельминтами.

Translated from English version into Russian by Oksana Weiss and Tatiana Com, through

TRANSLATORS
WITHOUT BORDERS

Los helmintos de importancia veterinaria y zoonótica en los rumiantes nigerianos: un metaanálisis de 46 años (1970-2016) de su frecuencia y distribución.

Solomon Ngutor Karshima, Beatty-Viv Maikai, Jacob Kwada Paghi Kwaga

Resumen
Antecedentes: la industria ganadera juega un papel vital en la economía de Nigeria. Se trata de la mayor fuente de ingresos y sustento para la mayoría de los nigerianos establecidos en las áreas rurales y contribuye a alrededor del 5,2% del Producto Nacional Bruto (PNB). Los helmintos, sin embargo, causan pérdidas económicas debido a las reducciones en la producción de leche, el aumento de peso, la fertilidad y la calidad de los cuerpos de los animales. Los helmintos zoonóticos de origen ganadero causan problemas de salud en humanos.
Métodos: El uso de las pautas de los Ítems de Informe Preferidos para Evaluaciones Sistemáticas y Meta-Análisis (PRISMA), la frecuencia y distribución de los helmintos de importancia veterinaria y zoonótica en los rumiantes nigerianos fueron determinados en un metaanálisis de datos publicados entre 1970 y 2016. Se estratificaron los datos en base a las regiones, anfitriones, períodos de estudio,

tamaño de la muestra y tipo de estudio mientras los helmintos fueron filogenéticamente agrupados en cestodos, nematodos y trematodos.

Resultados: Datos de 44 estudios llevados a cabo en 19 estados nigerianos revelaron una estimación de frecuencia agrupada (EFA) general del 7,48% (95% CI: 7,38-7,57) para los helmintos de importancia veterinaria y zoonótica de un total de 320.208 rumiantes. Observamos una variación significativa (p<0,001) entre las EFAs rango de 1,90% (95% CI: 1,78-2,02) y 60,98% (95% CI: 58,37-63,55) presentada a través de diferentes estratos. Una alta heterogeneidad (99,78%, 95% CI: 7,38-7,57) fue observada. *La estrongiloides papilar* fue la más frecuente (Frec: 32,02%, 95% CI: 31,01-33,11), mientras, *Fasciola gigantica* tuvo la distribución geográfica más amplia.

Conclusión: Los helmintos de importancia veterinaria y zoonótica son frecuentes en los rumiantes y están bien distribuidos en toda Nigeria Nuestras conclusiones muestran que los helmintos en los rumiantes pueden ser también posibles causantes de morbilidad en humanos y de pérdidas económicas en la industria ganadera en Nigeria. Una alta heterogeneidad se identificó entre los estudios y los diferentes estratos. Buenas prácticas agrícolas en las granjas, inspecciones veterinarias estándar de la carne, higiene adecuada y saneamiento de los mataderos, granjas y mercados de ganado necesitan implementarse en Nigeria a fin de reducir las amenazas económicas, de salud pública y veterinarias a causa de estos helmintos.

Translated from English version into Spanish by Victoria Miranda and Marina Cruselle, through

TRANSLATORS
WITHOUT BORDERS
