# Supplementary material for: Helminths of veterinary and zoonotic importance in Nigerian ruminants: a 46-year meta-analysis (1970–2016) of their prevalence and distribution
Source: Infect Dis Poverty. 2018 May 29;7:52. doi: 10.1186/s40249-018-0438-z (PMC5972411; doi:10.1186/s40249-018-0438-z)
Supplement: Supplementary file 3 — List of studies excluded from the meta-analysis. (DOCX 19 kb) [file 40249_2018_438_MOESM3_ESM.docx]

**LIST OF STUDIES EXCLUDED FORM THE ANALYSIS**

1. Adamu NB, Garba AE, Yazah AJ. Bacterial and parasitic zoonoses encountered at slaughter in Maiduguri abattoir, North-eastern Nigeria. Vet Wld, 2011;4(10):437-443.
2. [Bolajoko MB](https://www.ncbi.nlm.nih.gov/pubmed/?term=Bolajoko%20MB%5BAuthor%5D&cauthor=true&cauthor_uid=23163980), [Morgan ER](https://www.ncbi.nlm.nih.gov/pubmed/?term=Morgan%20ER%5BAuthor%5D&cauthor=true&cauthor_uid=23163980). Relevance of improved epidemiological knowledge to sustainable control of *Haemonchus contortus* in Nigeria. [Anim Hlth Res Rev.](https://www.ncbi.nlm.nih.gov/pubmed/23163980) 2012;13(2):196-208. doi: 10.1017/S1466252312000163.
3. Danbirni S, Ziyauhaq H, Allam L, Okaiyeto SO, Sackey AKB. Prevalence of liver condemnation due to fascioliasis in slaughtered cattle and it’s financial losses at Kano old abattoir, Nigeria. J Vet Adv. 2015;5(6):1004-1009. doi: 10.5455/jva.20150623092317.
4. Ejeh EF, Paul BT, Lawan FA, Lawal JR, Ejeh SA, Hambali IU. Seasonal prevalence of bovine fasciolosis and its direct economic losses due to liver condemnation at Makurdi abattoirs North-entral Nigeria. Sok J Vet Sci. 2015;13(2):42-48. <http://dx.doi.org/10.4314/sokjvs.v13i2.7>.
5. Ekong PS, Juryit R, Dika NM, Nguku P, Musenero M. Prevalence and risk factors for zoonotic helminth infection among humans and animals - Jos, Nigeria, 2005-2009. Pan Afr Med J. 2012;12(6): 6 pages, <http://www.panafrican-med-journal.com/content/article/12/6/full/>.
6. [Fagbemi BO](https://www.ncbi.nlm.nih.gov/pubmed/?term=Fagbemi%20BO%5BAuthor%5D&cauthor=true&cauthor_uid=6880006), [Dipeolu OO](https://www.ncbi.nlm.nih.gov/pubmed/?term=Dipeolu%20OO%5BAuthor%5D&cauthor=true&cauthor_uid=6880006). Moniezia infection in the dwarf breeds of small ruminants in Southern Nigeria. [Vet Q.](https://www.ncbi.nlm.nih.gov/pubmed/6880006) 1983;5(2):75-80.
7. [Fakae BB](https://www.ncbi.nlm.nih.gov/pubmed/?term=Fakae%20BB%5BAuthor%5D&cauthor=true&cauthor_uid=2247944). The epidemiology of helminthosis in small ruminants under the traditional husbandry system in eastern Nigeria. [Vet Res Commun.](https://www.ncbi.nlm.nih.gov/pubmed/2247944) 1990;14(5):381-91.
8. Ngele KK, Ibe E. Prevalence of fasciolopsis on cattle slaughtered at Eke market abattoir Afikpo, Ebonyi State, Nigeria. Nig J Parasitol. 2014;35(1-2):53-57.
9. Okoye IC, Obiezue RN, Okoye DN, Awi M. High prevalence of gastro-intestinal parasites in indigenous goats of Nigeria. Biotech. 2013;2(10);17-19.
10. Oladele-Bukola MO, Odetokun IA. Prevalence of bovine fasciolosis at the Ibadan municipal abattoir, Nigeria. Afr J Food Agric Nutr Dev. 2014;14(4):9055-9070.
11. [Onah DN](https://www.ncbi.nlm.nih.gov/pubmed/?term=Onah%20DN%5BAuthor%5D&cauthor=true&cauthor_uid=3759354), [Chiejina SN](https://www.ncbi.nlm.nih.gov/pubmed/?term=Chiejina%20SN%5BAuthor%5D&cauthor=true&cauthor_uid=3759354). *Taenia saginata* cysticercosis in slaughter cattle in Anambra State, Nigeria. [Intern J Zoon.](https://www.ncbi.nlm.nih.gov/pubmed/3759354) 1986;13(1):32-39.
12. Saulawa MA, Magaji AA, Faleke OO, Mohammed AA, Junaidu AU, Salihu MD, et al. Serodiagnosis of hydatidosis in sheep slaughtered at Sokoto abattoir, Sokoto state, Nigeria. Sok J Vet Sci. 2011;9(2):20-23.
13. Umeanaeto PU, Ogbogu NE, Irikannu KC, Onyido AE, Okwelogu IS, Mbanefo EC, Ifeanyichukwu MO. A comparative analysis of the gastro-intestinal helminth parasites of cattle in Awka and Obosi abattoirs in Anambra State, Southeastern Nigeria. J Adv Res Hlth Nurs. 2016;1(8): 6 pages.
